# Comparison of constant load exercise intensity for verification of maximal oxygen uptake following a graded exercise test in older adults

**DOI:** 10.14814/phy2.15037

**Published:** 2021-09-23

**Authors:** Ian R. Villanueva, John C. Campbell, Serena M. Medina, Theresa M. Jorgensen, Shannon L. Wilson, Siddhartha S. Angadi, Glenn A. Gaesser, Jared M. Dickinson

**Affiliations:** ^1^ Arizona State University Phoenix Arizona USA; ^2^ Department of Kinesiology University of Virginia Charlottesville Virginia USA; ^3^ Department of Health Sciences Central Washington University Ellensburg Washington USA

**Keywords:** aerobic power, aging, exercise physiology, verification, VO_2_max, VO_2_peak

## Abstract

Maximal oxygen uptake (VO_2_max) declines with advancing age and is a predictor of morbidity and mortality risk. The purpose here was to assess the utility of constant load tests performed either above or below peak work rate obtained from a graded exercise test for verification of VO_2_max in older adults. Twenty‐two healthy older adults (9M, 13F, 67 ± 6 years, BMI: 26.3 ± 5.1 kg·m^−2^) participated in the study. Participants were asked to complete two experimental trials in a randomized, counterbalanced cross‐over design. Both trials (cycle ergometer) consisted of (1) an identical graded exercise test (ramp) and (2) a constant load test at either 85% (CL85; *n* = 22) or 110% (CL110; *n* = 20) of the peak work rate achieved during the associated ramp (performed 10‐min post ramp). No significant differences were observed for peak VO_2_ (L·min^−1^) between CL85 (1.86 ± 0.72; *p* = 0.679) or CL110 (1.79 ± 0.73; *p* = 0.200) and the associated ramp (Ramp85, 1.85 ± 0.73; Ramp110, 1.85 ± 0.57). Using the study participant's mean coefficient of variation in peak VO_2_ between the two identical ramp tests (2.9%) to compare individual differences between constant load tests and the associated ramp revealed 19/22 (86%) of participants achieved a peak VO_2_ during CL85 that was similar or higher versus the ramp, while only 13/20 (65%) of participants achieved a peak VO_2_ during CL110 that was similar or higher versus the ramp. These data indicate that if a verification of VO_2_max is warranted when testing older adults, a constant load effort at 85% of ramp peak power may be more likely to verify VO_2_max as compared to an effort at 110% of ramp peak power.

## Graphical abstract

The intensity at which a constant load test can be used following a graded exercise test to verify maximal values in healthy older adults was examined. When verification of maximal values is warranted, our findings indicate that a constant load test performed at 85% of ramp peak power is more likely to verify a maximal effort and VO_2_max in healthy older adults as compared to a constant load effort at 110% of ramp peak power.

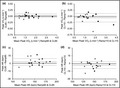

## 1 INTRODUCTION

Advancing age is associated with a variety of physiological and biological changes that can contribute to impaired physical function. Of particular interest is the steady decline in the maximal rate of oxygen uptake (VO_2_max) that is well documented during advancing age (Betik & Hepple, 2008; Gries et al., 1985; Kaminsky et al., 2015). Not only is a reduced VO_2_max in older adults associated with functional limitations, such as difficulty with walking, climbing stairs, and performing daily activities (Kaminsky et al., 2013; Paterson et al., 1999, 2004; Paterson & Warburton, 2010), but a low VO_2_max is a powerful independent predictor of cardiovascular disease and all‐cause mortality (Imboden et al., 2018; Kokkinos et al., 2010; Myers et al., 2002; Ross et al., 2016). Moreover, the addition of VO_2_max to other traditional risk factors improves risk stratification, and inclusion of VO_2_max to classify morbidity and mortality risk may be particularly powerful for those on the lower end of the VO_2_max spectrum, such as older adults (Ross et al., 2016). Consequently, developing effective exercise testing strategies that can be used to verify a maximal exercise effort, and thus VO_2_max, in older adults could have important implications for accurate assessment of morbidity and mortality risk in this population.

Traditionally, VO_2_max is often assessed through the use of a graded exercise test, employing either a steady ramp or an incremental step test until volitional exhaustion. In theory, a VO_2_max is achieved when there is no increase in VO_2_ with a concomitant increase in power or speed (Day et al., 2003; Hill & Lupton, 1923; Taylor et al., 1955), which is often referred to as a VO_2_ plateau (Taylor et al., 1955). While sampling rate/interval can influence the occurrence of a plateau in VO_2_ (Astorino, 2009)_,_ a plateau is not always observed, and in fact has been found to only occur in 17% of VO_2_max assessments (Day et al., 2003). The absence of an observed VO_2_ plateau has produced queries as to the validatity of these tests for accurately assessing VO_2_max (Day et al., 2003; Howley et al., 1995; Midgley & Carroll, 2009; Poole et al., 2008). Consequently, the development of secondary criteria that are predicated on expected values for respiratory exchange ratio, heart rate (HR), and blood lactate, for example, have been used to validate a maximal effort (Howley et al., 1995; Midgley et al., 2007, 2009; Wagner et al., 2020). However, the use of these secondary criteria is problematic (Poole & Jones, 2017) as these criteria can often be achieved at a “submaximal” effort (Poole et al., 2008).

More recently, the use of a secondary constant load test that is performed following a graded exercise test has been implemented as a strategy to verify a maximal effort and VO_2_max (Costa et al., 2021; Midgley & Carroll, 2009; Poole et al., 2008). While the use of a constant load test to verify VO_2_max has gained consideration, the intensity at which these constant load tests have been performed is variable. For instance, these constant load bouts have been performed at work rates below (Day et al., 2003; Murias et al., 2018; Rossiter et al., 2006; Sedgeman et al., 2013), equal to (Sawyer et al., 2015), or above (Astorino et al., 2009; Barker et al., 2011; Hawkins et al., 2007; Iannetta et al., 2020; Kuffel et al., 2005; Leicht et al., 2013; Midgley et al., 2006; Murias et al., 2018; Nolan et al., 2014; Poole et al., 2008; Rossiter et al., 2006; Scharhag‐Rosenberger et al., 2011; Sedgeman et al., 2013; Weatherwax et al., 2016) those achieved during the preceding graded exercise test [most previous studies employ constant load tests between 85% and 115% of peak work rate (Astorino et al., 2009; Barker et al., 2011; Dalleck et al., 2012; Hawkins et al., 2007; Iannetta et al., 2020; Kuffel et al., 2005; Leicht et al., 2013; Midgley & Carroll, 2009; Midgley et al., 2006; Murias et al., 2018; Niemela et al., 1980; Nolan et al., 2014; Poole et al., 2008; Rossiter et al., 2006; Sawyer et al., 2015; Scharhag‐Rosenberger et al., 2011; Sedgeman et al., 2013; Weatherwax et al., 2016)]. Furthermore, there is lack of agreement on the work rate at which the constant load tests should be performed to best verify a maximal effort (Breda et al., 1985; Iannetta et al., 2020; Poole & Jones, 2017). Specifically, some propose that if a VO_2_max is to be verified, the constant load effort needs to be conducted at a work rate higher than that achieved during the graded exercise test (Poole & Jones, 2017). On the other hand, recent evidence indicates that a work rate below that achieved during the graded exercise test is more likely to verify a maximal effort (Iannetta et al., 2020). In particular, the use of a “submaximal” constant load work rate to verify VO_2_max may be more reliable versus the “supramaximal” work rate when coupled with graded exercise test protocols that are shorter in duration (e.g., steeper ramp protocols) (Iannetta et al., 2020). Consequently, the use of a constant load work rate below the peak work rate achieved during the graded exercise test may be a more reliable strategy to verify VO_2_max in individuals with a lower VO_2_max, such as older adults, who may experience shorter graded exercise tests.

Therefore, the primary purpose of this study was to employ a cross‐over design to assess the utility of a constant load test performed at a work rate below (85%) and a work rate above (110%) the peak work rate achieved during a graded exercise test (ramp) for validating a maximal effort and verifying VO_2_max in healthy older adults. While comparison of constant load intensities above and below the peak achieved during a ramp test has been previously reported (Murias et al., 2018), to our knowledge no study has employed a randomized, counterbalanced cross‐over design. We hypothesized that in healthy older adults, the constant load test below the peak work rate of the ramp test would be more likely to verify a maximal effort and VO_2_max, which would be demonstrated by a greater number of individuals achieving a peak VO_2_ value during the constant load effort below peak work rate that is similar or higher to the ramp test as compared to the constant load effort above peak work rate. In addition, the randomized cross‐over design of the study included the performance of two identical ramp tests by each participant. Therefore, a secondary purpose of this study was to evaluate a second identical ramp test as a strategy to verify VO_2_max in older adults.

## 2 MATERIALS AND METHODS

### 2.1 Participants

Twenty‐four healthy older adults volunteered to participate in this study. All participants were between the ages of 60–80 years and were recruited by advertisement, locally posted flyers, and word of mouth. Participants completed a brief online pre‐screening questionnaire to assess general health characteristics which was reviewed by a member of the research team. Following the pre‐screening questionnaire, qualified participants were invited to the laboratory for a formal informed consent process. Additional screening included a medical history, the Physical Activity Readiness Questionnaire for Everyone (PARQ+), and assessment of resting blood pressure. Participants were excluded if they had uncontrolled hypertension, or any self‐reported heart, liver, kidney, blood, or respiratory disease, peripheral vascular disease, diabetes or endocrine disease, active cancer or use of tobacco, self‐reported acute or chronic illness, medical/orthopedic conditions precluding exercise, or if they were currently training for an endurance event (i.e., marathon, triathlon). All participants provided written informed consent prior to participation. Participant characteristics for those that participated in the study are presented in Table 1. This study was approved by the University Institutional Review Board (in compliance with the Declaration of Helsinki, as revised in 1983).

**TABLE 1:** Participant characteristics

	Men (*n* = 9)	Women (*n* = 13)	Total (*n* = 22)
Age, year	69 ± 6	65 ± 6	67 ± 6
Height, cm	172 ± 9	161 ± 5	165 ± 9
Weight, kg	77 ± 18	69 ± 16	72 ± 17
BMI, kg·m^−2^	26.0 ± 4.1	26.6 ± 5.8	26.3 ± 5.1
Body fat, %	28.1 ± 6.0	37.8 ± 10.5	34.0 ± 10.0
Lean body mass, kg	53 ± 12	39 ± 3	44 ± 10

Data are presented as mean ± SD.

Abbreviation: BMI, body mass index; Body fat % is whole body derived from dual energy x‐ray absorptiometry.

### 2.2 Study design and procedures

Participants were studied during two separate experimental trials. The experimental trials were separated on average by 9 days (range, 6–14 days) and were performed in a randomized, counterbalanced cross‐over design at a similar time of day (e.g., morning vs. afternoon). Each experimental trial consisted of a graded exercise ramp test and a constant load test that was performed after 10 min of active rest (pedaling at a work rate no higher than the warm‐up) following completion the ramp test. The ramp tests were identical for each experimental trial, however, the visits differed in the work rate at which the subsequent constant load test was performed.

During each experimental trial participants reported to the laboratory for testing at least 3 h postprandial and after abstaining from caffeine, alcohol, supplements, and exercise for at least 24 h. The participant's height and weight were measured on a calibrated stadiometer and resting blood pressure measures were obtained during each visit (Dinamap^®^ PRO 100 Vital Signs Monitor; GE Healthcare). Participants were equipped with a mouthpiece connected to a standard nonrebreathing valve (Hans Rudolph) for continuous measurement of ventilation and respiratory gas exchange data using a TrueOne 2400 metabolic cart (Parvomedics). A standard calibration was performed before each test per manufacturer recommendations. Participants were also equipped with a chest worn HR monitor (Polar, Inc.) to continuously monitor HR. After 2 min of rest, participants performed a standardized warm‐up in which the participants pedaled at a cadence of their choice, between 50 and 90 revolutions per minute (RPM), on a stationary cycle ergometer (Ergoline Viasprint 150) at 50 W for males and 40 W for females for 5 min. The chosen RPM was maintained for the remainder of the testing.

#### Ramp test

During both experimental trials, participants performed an identical ramp test on a cycle ergometer. Immediately following the warm‐up phase (described above), the work rate on the cycle ergometer was increased in a ramp fashion corresponding to 20 W·min^−1^ for males (1 W every 3 s) and 15 W·min^−1^ for females (1 W every 4 s) until volitional exhaustion. Ratings of perceived exertion (RPE) were assessed every 60 s throughout the duration of the ramp test. The test was terminated at volitional exhaustion or if the participant was unable to maintain his/her RPM despite verbal encouragement.

#### Constant load test

During each experimental trial a constant load test was completed following the ramp test, which occurred after 10 min of light active recovery (pedaling at a work rate no higher than the warm‐up) on the stationary ergometer. During active recovery, the participants were provided a break from the breathing valve, which was reconnected to the participant at least 3 min prior to the start of the constant load test. The constant load test consisted of cycling at a work rate equivalent to either 85% (CL85) or 110% (CL110) of the peak work rate reached during the preceding ramp test. Specifically, in a randomized, counterbalanced cross‐over design, participants were randomized to perform either CL85 or CL110 during the first visit, whereas during the second visit the participant completed the constant load test at the other work rate. Participants were instructed to increase cadence as the resistance on the cycle ergometer increased from that during active recovery to the prescribed intensity. Both constant load tests were performed at a constant work rate until volitional exhaustion. RPE was assessed at the end of the constant load test. The test was terminated at volitional exhaustion (i.e., the participant requesting to stop) or if the participant was unable to maintain his/her RPM despite verbal encouragement.

#### Assessment of body composition

During the second visit, participants underwent a dual‐energy x‐ray absorptiometry (DEXA) whole‐body scan (Lunar iDXA, GE Healthcare). The DEXA was performed prior to any testing and after voiding the bladder. Participants laid down on the DEXA for 15 min prior to the DEXA to avoid any influence of fluid shifts. A trained and certified radiologist administered the DEXA scan.

### 2.3 Assessment of physiological outcomes

All ventilation and gas exchange data were assessed using 10‐s average measurements, with O_2_ and CO_2_ concentration of expired air derived from samples obtained from a mixing chamber. Peak VO_2_ values for the ramp tests and constant load tests were taken as the highest three consecutive 10‐s measurements, which were averaged to yield data collected over a 30‐s timeframe. Peak RER values were taken as an average of the three 10‐s measurements at the same time point as peak VO_2_. Peak HR for the ramp and constant load tests were taken as the highest recorded HR. Peak power during the ramp was identified as the highest work rate achieved prior to a drop in cadence or volitional exhaustion. Individual data were calculated to determine the percent change of physiological outcomes between the constant load test and the associated ramp, as well as between the ramp during the first visit (Ramp1) compared to the ramp during the second visit (Ramp2). The mean coefficient of variation (CV) for Ramp1 and Ramp2 was used to identify if a similar (within CV) or a higher or lower value (outside CV) for a physiological variable occurred between the constant load test and the associated ramp test and between Ramp1 and Ramp2.

### 2.4 Statistical analysis

All data were tested for normality through skewness and kurtosis analyses and visual inspection of the normality plots using SPSS v.24 (IBM). A one‐way, repeated measures, analysis of variance (ANOVA) was used to assess differences between the ramp tests and the constant load tests for all outcomes. Pairwise comparisons were performed following the ANOVA using a least significant difference (LSD) post hoc analyses adjusted for the following two comparisons: constant load test at 85% of peak work rate (CL85) versus the associated ramp (Ramp85); and constant load test at 110% of peak work rate (CL110) versus the associated ramp (Ramp110). Outcome variables obtained from the first (Ramp1) and second ramp test (Ramp2) were compared using a dependent *t*‐test for equivalence. Pearson's correlations were used to determine the relationships between variables for the constant load test versus ramp and for Ramp1 versus Ramp2. Bland–Altman plots and CVs were used to compare the agreement for all variables between the constant load test and associated ramp test and between Ramp1 and Ramp2. Intraclass correlation coefficients (ICCs) were used to examine the reliability of peak VO_2_ and peak HR between the constant load test and associated ramp test and between Ramp1 and Ramp2. All comparisons including a constant load test were made to the ramp performed during the same experimental trial. Pearson's correlations were also used to examine the following relationships within each experimental trial: (1) Difference in peak VO_2_ (L·min^−1^) between CL85 and Ramp85 and time to exhaustion for CL85, (2) Difference in peak VO_2_ (L·min^−1^) between CL85 and Ramp85 and time to exhaustion of Ramp85, (3) Difference in peak VO_2_ (L·min^−1^) between CL110 and Ramp110 and time to exhaustion of CL110, and (4) Difference in peak VO_2_ (L·min^−1^) between CL110 and Ramp110 and time to exhaustion during Ramp110. All data were analyzed using SPSS Software (SPSS v24) and significance was set a priori at *p* ≤ 0.05. All data are presented as means ± SD.

## 3 RESULTS

Of the 24 participants who enrolled in the study, two participants were excluded during the screening process (one for high blood pressure, one for underlying medical disease). Two additional participants dropped out of the study after completing the first visit due to circumstances unrelated to the study. Both of these participants only completed the CL85 exercise trial, and these participants were included in the analysis for ramp versus CL85 (e.g., CL85, *n* = 22; CL110, *n* = 20). Only participants who completed both trials (*n* = 20; 67 ± 6 year, 8 males and 12 females) were included in the comparisons between Ramp1 and Ramp2. Participant characteristics are presented in Table 1. In addition, the peak VO_2_ (mLO_2_·kg^−1^·min^−1^) and peak HR of these participants are expressed relative to age‐based reference standards (Kaminsky et al., 2015) in Table 2.

**TABLE 2:** Study participants relative peak VO_2_ (mLO_2_·kg^−1^·min^−1^) and heart rate (HR) in comparison to reference standards derived from FRIEND (Kaminsky et al., 2015)

	Study participants	Reference	Percentile
Males			
Peak VO_2_ (mlO_2_·kg^−1^·min^−1^)	29.8 ± 9.6 (18.5–49.9)	29.4 ± 7.9	~50th
Peak HR (bpm)	159 ± 17 (135–186)	158 ± 17	N/A
Females			
Peak VO_2_ (mLO_2_·kg^−1^·min^−1^)	24.2 ± 10.5 (14.1–47.9)	20.7 ± 5.0	~75th
Peak HR (bpm)	147 ± 17 (120–175)	157 ± 17	N/A

Study Participant data (9M, 13F, 67 ± 6 years) are presented as mean ± SD (range) from the first visit ramp test. Reference and percentile data are derived from FRIEND for age 60–69 years (Kaminsky et al., 2015).

Abbreviation: bpm, beats per minute.

### 3.1 Ramp versus constant load test (group data)

**FIGURE 1:**
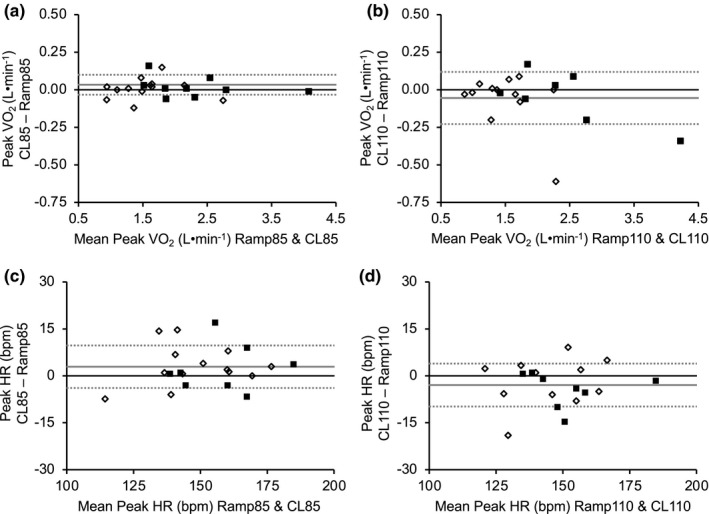
Bland–Altman plots for peak oxygen uptake (VO_2_, L·min^−1^) and heart rate (HR). Presented are (a) peak VO_2_ obtained during the constant load test performed at 85% of ramp peak work rate (CL85) and the associated ramp test (Ramp85), (b) peak VO_2_ obtained during the constant load test performed at 110% of ramp peak work rate (CL110) and the associated ramp test (Ramp110), (c) peak HR obtained during CL85 and Ramp85, and (d) peak HR obtained during CL110 and Ramp110. *Y*‐axis = constant load test − ramp; *x*‐axis = mean of ramp and constant load test; dotted lines = mean ± 1.96 × SD; dark solid lines = 0 on the *y*‐axis; light solid lines = mean of constant load test − ramp. Filled squares (■) represent male participants and open diamonds (♢) represent female participants. Ramp85 versus CL85, *n* = 22; Ramp110 versus CL110, *n* = 20

**FIGURE 2:**
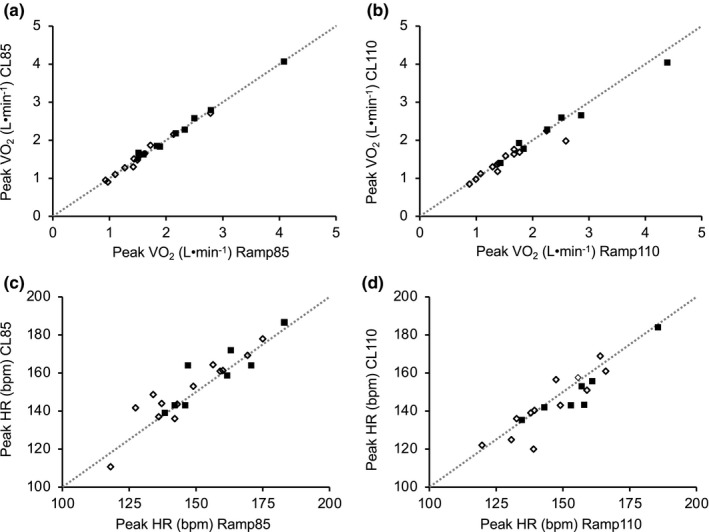
Peak oxygen uptake (VO_2_, L·min^−1^) and heart rate (HR) achieved during the ramp (*x*‐axis) and constant load (*y*‐axis) test for each participant. The dotted lines represent the line of identity (*y* = *x*). Presented are (a) peak VO_2_ obtained during the constant load test at 85% of ramp peak work rate (CL85) versus the associated ramp (Ramp85), (b) peak VO_2_ obtained during the constant load test at 110% of ramp peak work rate (CL110) versus the associated ramp (Ramp110), (c) peak HR obtained during CL85 versus Ramp85, and (d) peak HR obtained during CL110 versus Ramp110. Filled squares (■) represent male participants and open diamonds (♢) represent female participants. Ramp85 versus CL85, *n* = 22; Ramp110 versus CL110, *n* = 20

**FIGURE 3:**
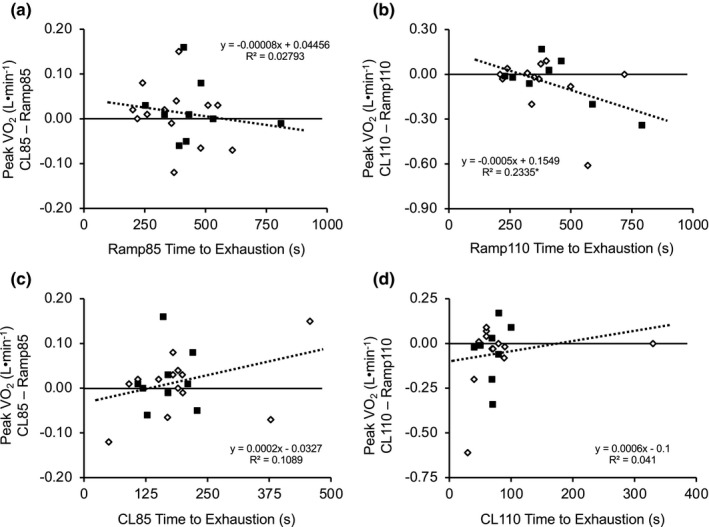
Correlations between time to exhaustion (*x*‐axis) and differences in peak oxygen uptake (VO_2_, L·min^−1^) achieved during the constant load and ramp tests. Presented are (a) time to exhaustion during the associated ramp (Ramp85) compared to the difference in peak VO_2_ obtained during the constant load test at 85% of ramp peak power (CL85) and Ramp85, (b) time to exhaustion during the associated ramp (Ramp110) compared to the difference in peak VO_2_ obtained during the constant load test at 110% of ramp peak power (CL110) and Ramp110, (c) time to exhaustion during CL85 compared to the difference in peak VO_2_ obtained during CL85 and Ramp85, and (d) time to exhaustion during CL110 compared to difference in peak VO_2_ obtained during CL110 and Ramp110. *p < 0.05. Filled squares (■) represent male participants and open diamonds (♢) represent female participants. Ramp85 versus CL85, *n* = 22; Ramp110 versus CL110, *n* = 20

Peak VO_2_ (L·min^−1^) did not differ (*p* = 0.679) between Ramp85 (1.85 ± 0.73 L·min^−1^) and CL85 (1.86 ± 0.72 L·min^−1^) (CV = 2.07 ± 2.14%) (Table 3, Figures 1a and 2a). Similarly, peak VO_2_ was not significantly different (*p* = 0.200) between Ramp110 (1.85 ± 0.57 L·min^−1^) and CL110 (1.79 ± 0.73 L·min^−1^) (CV =3.64% ± 4.47%) (Table 3, Figures 1b and 2b). Intraclass correlations also showed agreement in peak VO_2_ (L·min^−1^) between the ramp and constant load test for both CL85 (ICC = 0.997) and CL110 (ICC = 0.979) (Table 3). Time to exhaustion during the ramp and constant load tests were examined to determine whether time to exhaustion of the various tests influenced differences in peak VO_2_ between the constant load test and associated ramp. Time to exhaustion for Ramp85 was not statistically correlated with the difference in peak VO_2_ between CL85 and Ramp85 (*r* = 0.17; *p* = 0.458) (Figure 3a). However, a longer Ramp110 time to exhaustion was negatively associated with the difference in peak VO_2_ between CL110 and Ramp110 (*r* = 0.48; *p* = 0.031) (Figure 3b), indicating that a longer time to exhaustion during the ramp test was associated with a greater likelihood of attaining a lower peak VO_2_ during CL110 compared to the ramp. Time to exhaustion for the respective constant load test protocols was not statistically correlated with the difference in peak VO_2_ between CL85 and the associated ramp (*r* = 0.33; *p* = 0.134) (Figure 3c) or between CL110 and the associated ramp (*r* = 0.20; *p* = 0.393) (Figure 3d).

**TABLE 3:** Physiological group and individual responses to the ramp and constant load tests

	Peak VO_2_ (L·min^−1^)	Peak HR (bpm)	VE (L·min^−1^)	Peak RER	Power (W)	Time to exhaustion (s)
CV (%)^a^	2.9	2.3	6.3	3.2	5.3	8.0
Ramp 1 versus Ramp 2						
Ramp1	1.82 ± 0.72	150 ± 17	76.91 ± 31.69	1.16 ± 0.09	156 ± 53	402 ± 151
Ramp2	1.86 ± 0.81	149 ± 15	79.31 ± 31.84	1.16 ± 0.08	158 ± 53	408 ± 160
Ind. Similar^b^	(9/20)	(9/19)	(9/20)	(7/20)	(9/20)	(9/20)
Ind. Higher^b^	(8/20)	(3/19)	(6/20)	(5/20)	(7/20)	(6/20)
Ind. Lower^b^	(3/20)	(7/19)	(5/20)	(8/20)	(4/20)	(5/20)
Ramp versus constant load test at 85%						
Ramp	1.85 ± 0.73	150 ± 17	77.85 ± 30.01	1.17 ± 0.09	158 ± 52	401 ± 142
CL85	1.86 ± 0.72	153 ± 17	80.15 ± 30.20	1.07 ± 0.08*	133 ± 45	185 ± 88
Ind. Similar^b^	(15/22)	(11/21)	(10/22)	(1/22)		
Ind. Higher^b^	(4/22)	(7/21)	(8/22)	(2/22)		
Ind. Lower^b^	(3/22)	(3/21)	(4/22)	(19/22)		
Ramp versus constant load test at 110%						
Ramp	1.85 ± 0.57	149 ± 16	78.28 ± 33.63	1.16 ± 0.08	156 ± 54	410 ± 162
CL110	1.79 ± 0.73	146 ± 16	75.83 ± 34.83	1.03 ± 0.10*	170 ± 60	79 ± 62
Ind. Similar^b^	(8/20)	(7/19)	(9/20)	(2/20)		
Ind. Higher^b^	(5/20)	(3/19)	(6/20)	(0/20)		
Ind. Lower^b^	(7/20)	(9/19)	(5/20)	(18/20)		

Data are presented as mean ± SD.

^a^ Mean individual participant coefficient of variation (CV) from Ramp1 to Ramp2, presented as percent (%).

^b^ Number of participants with values within the CV (for Ramp1 to Ramp2) between tests (similar), a value that is identified as higher (>CV for Ramp1 to Ramp2) compared to the Ramp (or compared to Ramp 1 for Ramp2 vs. Ramp1) (higher), a value that is identified as lower (>CV for Ramp1 to Ramp2) compared to the Ramp (or compared to Ramp 1 for Ramp2 vs. Ramp1) (lower). HR, heart rate; RER, respiratory exchange ratio; VE, ventilation; Ind. Similar, represents the number of participants that achieved a similar value (within CV) during the constant load test versus the associated ramp or for Ramp2 versus Ramp1; Ind. Higher, represents the number of participants that achieved a higher value (outside CV) during the constant load (CL) test versus the associated ramp or for Ramp2 versus Ramp1; Ind. Lower, represents the number of participants that achieved a lower value (outside CV) during the constant load test versus the associated ramp or for Ramp2 versus Ramp1.

* *p* < 0.05 Ramp.

Peak HR did not differ (*p* = 0.243) between Ramp85 (150 ± 17 bpm) and CL85 (153 ± 17 bpm) (Table 3, Figures 1c and 2c). Similarly, peak HR did not differ (*p* = 0.085) between Ramp110 (149 ± 16 bpm) and CL110 (146 ± 16 bpm) (Table 3, Figures 1d and 2d). Intraclass correlations showed agreement in peak HR between ramp and constant load test for both CL85 (ICC = 0.950) and CL110 (ICC = 0.906). Peak RER was significantly different (*p* < 0.01) between Ramp85 (1.17 ± 0.09) and CL85 (1.07 ± 0.08) (Table 3). Similarly, peak RER was significantly different (*p* < 0.01) between Ramp110 (1.16 ± 0.08) and CL110 (1.03 ± 1.0) (Table 3). Peak RPE did not differ (*p* = 0.602) between Ramp85 (18.5 ± 1.3) and CL85 (18.3 ± 1.7). Similarly, peak RPE did not differ (*p* = 0.629) between Ramp110 (18.7 ± 1.0) and CL110 (18.6 ± 1.1).

### 3.2 Ramp1 versus Ramp2 (group data)

**FIGURE 4:**
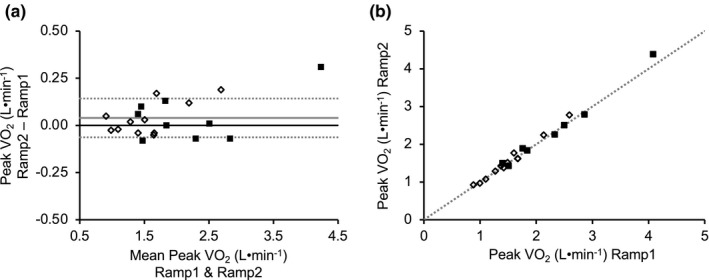
Comparison of peak VO_2_ values (L·min^−1^) achieved during the first ramp test (Ramp1) and the second ramp test (Ramp2). Presented are (A) Bland–Altman plot for peak VO_2_ obtained during Ramp1 and Ramp2 [*Y*‐axis = Ramp2 − Ramp1; *x*‐axis = mean of Ramp1 and Ramp2; dotted lines = mean ± 1.96 × SD; dark solid lines = 0 on the *y*‐axis; light solid lines = mean of Ramp1 − Ramp2] and (b) the relationship between peak VO_2_ obtained during Ramp1 and Ramp2 [the line represents the line of identity (*y* = *x*)]. Filled squares (■) represent male participants and open diamonds (♢) represent female participants (*n* = 20)

Peak VO_2_ during Ramp1 (1.82 ± 0.72 L·min^−1^) was not significantly different (*p* = 0.100) from Ramp2 (1.86 ± 0.81 L·min^−1^) (CV = 2.90 ± 1.89%) (Table 3). Peak VO_2_ was also strongly correlated (*R* ^2^ = 0.987) (*p* < 0.01) and was in high agreement (ICC = 0.994) between Ramp1 and Ramp2 (Figure 4). Peak HR did not differ (*p* = 0.115) between Ramp1 (150 ± 17 bpm) and Ramp2 (149 ± 15 bpm) (CV = 2.30 ± 2.06%) (Table 3) and values were strongly correlated (*R* ^2^ = 0.876) (*p* < 0.01) and in high agreement (ICC = 0.936) between Ramp1 and Ramp2. RER did not differ (*p* = 0.348) between Ramp1 (1.16 ± 0.09) and Ramp2 (1.16 ± 0.08) (CV = 3.20 ± 2.05%) (Table 3) and values were correlated (*R* ^2^ = 0.529) (*p* < 0.01) (Table 3) and in agreement (ICC = 0.727). Peak power output (W) did not differ between Ramp1 (156 ± 53) and Ramp2 (158 ± 53) (CV = 5.3 ± 5.40%) (Table 3) and values were strongly correlated (*R* ^2^ = 0.905) (*p* < 0.01) and in high agreement (ICC = 0.951) between Ramp1 and Ramp2. RPE did not differ (*p* = 0.481) between Ramp1 (18.5 ± 1.1) and Ramp2 (18.6 ± 1.3) and values were correlated (*R* ^2^ = 0.480) (*p* < 0.01).

### 3.3 Individual data

We calculated the mean individual participant CV (%) between Ramp1 and Ramp2 to examine individual differences in physiological variables between the ramps (Ramp1 vs. Ramp2) and between the constant load tests and their associated ramp tests. Using this participant‐based CV‐derived cut point from Ramp1 and Ramp2 (see Table 3), 68% of participants (15 of 22) achieved a peak VO_2_ during CL85 that was similar (within 2.9%, CV between Ramp1 and Ramp2 for peak VO_2_) to the associated ramp peak VO_2_. Furthermore, 18% of participants (4 of 22) achieved a peak VO_2_ during CL85 that was >2.9% higher than that achieved during Ramp85, while 14% of participants (3 of 22) achieved a peak VO_2_ during CL85 that was >2.9% lower than that achieved during Ramp85 (Table 3). In contrast, 40% of participants (8 of 20) achieved a peak VO_2_ during CL110 that was similar to the associated ramp, 25% of participants (5 of 20) achieved a peak VO_2_ during CL110 that was >2.9% higher than Ramp110 (Table 3), and 35% of participants (7 of 20) achieved a peak VO_2_ during CL110 that was >2.9% lower than Ramp110. Similar results were observed between CL85 and CL110 for peak HR and ventilation (Table 3).

When comparing Ramp2 to Ramp1, 45% of participants (9 of 20) achieved a peak VO_2_ during Ramp2 that was similar to Ramp1, 40% of participants (8 of 20) achieved a peak VO_2_ during Ramp2 that was identified as higher (>2.9% difference) compared to Ramp1, and 15% (3 of 20) achieved a peak VO_2_ during Ramp2 that was identified as lower (>2.9% difference) compared to Ramp1 (Table 3). Results for peak HR, VE, and RER are also presented in Table 3.

We recognize the lack of consensus on methodological/statistical approaches for confirming VO_2_max during a constant load (verification) test (or any secondary test). Therefore, Table 4 provides additional information on individual differences/similarities between tests using ±2 × typical error of the two ramp tests (McCarthy et al., 2021) and a HR of ±2 bpm (Midgley et al., 2006) or ±4 bpm (Midgley et al., 2009) from the peak HR from the ramp tests. In all instances (study CV, ±2 × typical error, HR ±2 or ±4 bpm), when compared to CL110, CL85 had a greater percentage of individuals with a constant load test that was considered similar to or higher than the ramp.

**TABLE 4:** Comparison of various individual data “cut points” used in the literature to determine verification of VO_2_max

	Study CV (±2.9%)	2 × TE (±0.156 L·min^−1^)	Heart rate (±2 bpm)	Heart rate (±4 bpm)
Ramp 1 versus Ramp 2				
Ind. Similar^a^	(9/20)	(17/20)	(8/19)	(11/19)
Ind. Higher^a^	(8/20)	(3/20)	(2/19)	(1/19)
Ind. Lower^a^	(3/20)	(0/20)	(9/19)	(7/19)
Ramp versus constant load test at 85%				
Ind. Similar^a^	(15/22)	(21/22)	(10/21)	(12/21)
Ind. Higher^a^	(4/22)	(1/22)	(7/21)	(7/21)
Ind. Lower^a^	(3/22)	(0/22)	(4/21)	(2/21)
Ramp versus constant load test at 110%				
Ind. Similar^a^	(8/20)	(15/20)	(8/19)	(12/19)
Ind. Higher^a^	(5/20)	(1/20)	(6/19)	(4/19)
Ind. Lower^a^	(7/20)	(4/20)	(5/19)	(3/19)

^a^ The criteria for a similar, higher, or lower value were that the value had to be within or outside (±) the study coefficient of variation (CV), 2 × typical error (TE) (McCarthy et al., 2021), or a heart rate within 2 beats per minute (bpm) (Midgley et al., 2006) or 4 bpm (Midgley et al., 2009) of the peak heart rate achieved during the ramp. Ind. Similar, represents the number of participants that achieved a similar value (within cut points) during the constant load test versus the associated ramp or for Ramp2 versus Ramp1; Ind. Higher, represents the number of participants that achieved a higher value (outside cut point) during the constant load test versus the associated ramp or for Ramp2 versus Ramp1; Ind. Lower, represents the number of participants that achieved a lower value (outside cut point) during the constant load test versus the associated ramp or for Ramp2 versus Ramp1.

## 4 DISCUSSION

To our knowledge this is the first study to employ a randomized, counterbalanced cross‐over design to evaluate the utility of constant load tests performed above and below ramp‐derived peak work rate to serve as a strategy to verify a maximal effort and VO_2_max in healthy older adults. The primary finding from this investigation is that in healthy older adults, a constant load test performed at a work rate slightly below (85%) peak work rate achieved during a graded exercise test was more likely to verify VO_2_max as compared to a constant load test performed at a work rate above (110%) that achieved during a graded exercise test. In addition, our data also indicate that while a second identical ramp test could produce a slightly higher peak VO_2_ in a greater number of individuals as compared to the constant load test at 85% peak work rate, both strategies yield reasonably similar outcomes for verifying VO_2_max.

Relative to younger adults, little attention has been given to the efficacy of a constant load test for verifying a maximal effort and VO_2_max in older adults (Dalleck et al., 2012; Murias et al., 2018). In this study, we examined to what extent a constant load test performed above (110%) or below (85%) ramp peak work rate could be used to verify VO_2_max in healthy older adults. We specifically chose these work rates as they represent the range in intensity used in previous studies that used a constant load “verification” test (Astorino et al., 2009; Barker et al., 2011; Costa et al., 2021; Dalleck et al., 2012; Day et al., 2003; Kuffel et al., 2005; Midgley & Carroll, 2009; Murias et al., 2018; Niemela et al., 1980; Poole et al., 2008; Rossiter et al., 2006; Sawyer et al., 2015; Sedgeman et al., 2013). Consistent with many previous studies, we did not identify “group” differences for peak VO_2_ achieved between the ramp test and the corresponding constant load test, regardless of intensity. However, examination of the individual participant data revealed a greater likelihood for the CL85 test to validate a maximal effort and VO_2_max as compared to CL110. Specifically, only 3 of the 22 participants (~14%) achieved a peak VO_2_ during the CL85 that was lower (outside the CV of the two ramp tests) than the value achieved during the ramp test. These data indicate that ~86% of the participants (19 of 22) achieved a peak VO_2_ during the CL85 that was either similar (15 of 22 participants, within the CV of the two ramp tests) or higher (4 of 22 participants, >CV of the two ramp tests) than that achieved during the associated ramp test.

In contrast, 7 of 20 participants (~35%) achieved a peak VO_2_ during the CL110 test that was lower (>CV of the two ramp tests) than the value achieved during the ramp test, and thus only ~65% achieved a value that was similar (8 of 20 participants) or higher (5 of 20 participants) than the associated ramp test. While we acknowledge previously proposed rationale that the constant load “verification” test should, theoretically, be conducted at a work rate higher than that achieved during the ramp test (e.g., supramaximal) (Poole & Jones, 2017), the present results indicate that a constant load test performed at a work rate of 110% of ramp peak power may be too high for some older adults as a method to verify a maximal effort and VO_2_max. Moreover, the greater agreement in VO_2_peak between the ramp test and CL85 as compared to the ramp test and CL110 is also evident through examination of the limits of agreement and bias presented in the Bland–Altman plots (Figure 1a and 1b), as well as when employing other cut points used in the literature (see Table 4). Collectively, our findings further support (Iannetta et al., 2020) the use of a work rate slightly below peak ramp work rate, as opposed to above, when a constant load test to verity a maximal effort and VO_2_max in healthy older adults is warranted. Moreover, these results also further support the use of individual data for assessment of VO_2_max and comparison of constant load “verification” test intensities (Noakes, 2008).

As expected, the CL110 test elicited a shorter exercise duration (mean ~79 s [range, 30–330 s]) compared to CL85 (mean ~185 s [range, 50–457 s]). Previous research in older adults that used a constant load test at 105% of ramp peak work rate reported mean durations of ~102 s (Murias et al., 2018) and ~150 s (Dalleck et al., 2012). The shorter duration observed during CL110 in this study may be due to the 5% difference in constant load test work rate in participants of approximately the same age (Dalleck et al., 2012; Murias et al., 2018). It is also important to note that the greater likelihood of lower peak VO_2_ values during CL110 could be the result of a reduced contribution of the slow component of VO_2_ (Gaesser & Poole, 1996). Specifically, it has been reported that an exercise duration of >3 min is necessary to observe changes in VO_2_ kinetics that are due to the VO_2_ slow component (Gaesser & Poole, 1996). However, we did not observe any significant correlations between exercise time of the constant load test and agreement between peak VO_2_ achieved during the ramp and corresponding constant load test (Figure 3). Interestingly, we did observe that a longer time to exhaustion during Ramp110 (thus, higher peak power) was more likely to result in a lower peak VO_2_ during CL110. This finding would appear to agree with previous work suggesting that a peak VO_2_ achieved during a ramp protocol that resulted in a higher peak power was less likely to be validated with a constant load effort above the ramp peak power (Iannetta et al., 2020). To that end, with the exception of one participant who had a history of cycling (highest VO_2_max), participants were relatively unaccustomed to cycling exercise. Thus, the lower likelihood of verifying VO_2_max in these older adults when using a constant load test above ramp peak work rate may be due to an inability to tolerate the physiological demands of such high work rates for a sufficiently long enough time to elicit VO_2_max. This may also explain why nearly 50% (9 of 19) of the participants achieved a peak HR during CL110 that was lower (outside the CV of the two ramp tests) than that achieved during the associated ramp.

In this study, participants completed two identical ramp assessments approximately 1 week apart (mean = 9 days). We chose this time frame to provide adequate recovery time from the previous test. The mean CV observed for peak VO_2_ between the two ramp tests is consistent with ranges identified in previous reports (Fielding et al., 1997; Foster et al., 1986; Skinner et al., 1999), and as discussed above, we utilized the mean participant CV (%) from the two identical ramp tests to identify individual differences in physiological variables between ramp and constant load tests. The design of the study also allowed us to examine to what extent a second ramp test could be used to assess/verify VO_2_max in older adults. Consistent with previous reports (Foster et al., 1986), we did not observe any significant differences in any physiological variable between the first visit (Ramp1) and the second visit (Ramp2). In addition, using the CV‐derived cut point, the number of participants that achieved a similar or higher peak VO_2_ during Ramp2 compared to Ramp1 (17/20 participants) was similar to that observed when comparing CL85 to the ramp (19/22 participants). However, when compared to the ramp versus constant load test comparisons, more participants achieved a higher peak VO_2_ during Ramp2 compared to Ramp1 (40%; 8/20 participants). Importantly, these discrepancies in peak VO_2_ achieved during the ramp in the first and second experimental trial did not impact the comparison between the associated ramp and constant load tests. Not only was the study counter‐balanced, but among participants who completed both trials and achieved a peak VO_2_ during a constant load test that was different compared to the associated ramp test, there was a similar number of participants who achieved a different (higher or lower) peak VO_2_ during the constant load test during the first (higher value, *n* = 5; lower value, *n* = 4) and during the second experimental trial (higher value, *n* = 4; lower value, *n* = 6). Collectively, these data indicate that some individuals may not be accustomed to the maximal intensity of exercise, the mode of exercise, or perhaps the breathing apparatus (Poole & Jones, 2012). Moreover, the results of this study indicate that a familiarization trial or second ramp could also increase the accuracy of VO_2_max assessments in some older adults, perhaps for a slightly greater number of individuals as compared to the use of a constant load test.

Peak HR was not different during the ramp test and either constant load test intensity. This finding contrasts with the results of a previous study with older adults that found a significantly higher peak HR during a ramp test as compared to a supramaximal verification test (105%) and submaximal (85%) verification test (Murias et al., 2018), although the magnitude of difference in that study (Murias et al., 2018) was extremely small (1–2 bpm). Moreover, similar to VO_2_max discussed above, individual data indicate that a greater number of participants achieved a similar or higher peak HR during CL85 versus the ramp as compared to CL110 versus the ramp (86% vs. 53%). In addition, the individual data and visual inspection of the Bland–Altman plots suggest a greater likelihood for participants to achieve a lower peak HR during CL110 versus the ramp as compared to CL85. Together with the VO_2_ data, these peak HR data further support the incorporation of a constant load test performed slightly below peak ramp work rate for verification of maximal values in older adults.

We recognize that previous studies have utilized rest periods as short as 3 min and as long as a full week between ramp and constant load verification tests (Astorino et al., 2009; Barker et al., 2011; Dalleck et al., 2012; Day et al., 2003; Hawkins et al., 2007; Kuffel et al., 2005; Leicht et al., 2013; Midgley & Carroll, 2009; Midgley et al., 2006; Murias et al., 2018; Niemela et al., 1980; Nolan et al., 2014; Poole et al., 2008; Rossiter et al., 2006; Sawyer et al., 2015; Scharhag‐Rosenberger et al., 2011; Sedgeman et al., 2013; Weatherwax et al., 2016), and thus we cannot extend our findings to situations that may utilize different rest periods between tests. However, we specifically employed a 10‐min active rest period between the end of the ramp test and the initiation of the constant load test as this timeframe is likely to be more practical for future research and clinical practice as participants would not be required to come back for testing at a later time or date. In addition, it is possible that our findings may have been influenced by the duration of the ramp test (Iannetta et al., 2020). Similarly, some reports indicate that a valid VO_2_max is achieved with a ramp test of at least 8 min (Buchfuhrer et al., 1983), although this notion has been challenged (Midgley et al., 2008). Finally, we acknowledge that the necessity of verification tests has been questioned (Murias et al., 2018; Wagner et al., 2021), perhaps on the basis that a high percentage of verification tests yield peak VO_2_ values that are considered similar to the ramp tests. Indeed, if the graded exercise test was a maximal effort, then in theory the ramp and constant load tests should yield similar values. In addition, it is important to note that previous studies (see (Costa et al., 2021)), as well as data from the current investigation, demonstrate that not all ramp tests will yield maximal VO_2_ values (or values that are similar between the ramp and secondary verification test). Importantly, those ramp efforts that do and do not produce maximal values could not be identified without employing a secondary test to verify the results. Future investigators and/or clinicians will need to determine, for their specific use, the necessity to obtain an accurate measurement of VO_2_max and to what extent a value requires “verification” using a single visit or multiple visit approach.

In conclusion, these findings have implications for the evaluation of VO_2_max of older adults in both a research and clinical setting. In particular, given the overwhelming data to suggest VO_2_max/cardiorespiratory fitness is perhaps the most powerful predictor of cardiovascular disease risk (Kokkinos et al., 2010; Myers et al., 2002; Ross et al., 2016), identifying strategies to obtain an accurate assessment of VO_2_max in older adults will serve to better identify individuals at risk for cardiovascular disease as well as those with increased risk of morbidity and mortality. Specifically, our data indicate that when verification of maximal values is warranted in a single testing session, a constant load test performed at 85% of ramp peak power is more likely to verify a maximal effort and VO_2_max in older adults as compared to a constant load test at 110% ramp peak power. On the other hand, in situations where multiple participant visits are feasible, performing an additional ramp test may also serve to verify VO_2_max, and could potentially lead to higher values in a slightly greater number of participants. However, the logistics and associated participant burden of recovery times between tests in a single session and/or multiple visits must be considered in the application of constant load testing to verify VO_2_max in the real‐world settings (especially clinical environments and clinical populations).

## DISCLOSURES

The authors have no conflict of interest to declare.
